# Bibliometric analysis: Root and root canal morphology using cone‐beam computed tomography

**DOI:** 10.1002/cre2.801

**Published:** 2023-10-25

**Authors:** Mohmed Isaqali Karobari, Beenish Fatima Alam, Raima Bashir, Muhammed Faisal Fahim, Mubashir Baig Mirza, Tahir Yusuf Noorani

**Affiliations:** ^1^ Department of Restorative Dentistry & Endodontics, Faculty of Dentistry University of Puthisastra Phnom Penh Cambodia; ^2^ Department of Oral Biology Bahria University Dental College Karachi Pakistan; ^3^ Department of BUCPT Bahria University Health Sciences Karachi Pakistan; ^4^ Conservative Dental Science Department, College of Dentistry Prince Sattam bin Abdulaziz University Al‐Kharj Saudi Arabia; ^5^ Conservative Dentistry Unit, School of Dental Sciences Universiti Sains Malaysia Kota Bharu Kelantan Malaysia

**Keywords:** dental anatomy, dental diagnostic imaging, dental pulp, endodontics

## Abstract

**Objectives:**

This bibliometric analysis aimed to evaluate the leading nations, authors, journals, institutes, highly cited publications, and most commonly used keywords concerning scientific publications based on root and root canal morphology using the CBCT.

**Material and Methods:**

For this bibliometric analysis, an extensive search was carried out on September 25, 2023 using the Scopus database. Pertinent articles in the field were scrutinized after applying inclusion and exclusion criteria. Data were evaluated using Vosviewer and Microsoft Excel.

**Results:**

A literature search revealed the initiation of scientific publication in 2008. Significant contributions made by Saudi Arabia, India, and China concerning the number of publications were seen. Similarly, Jazan University from SA was recognized as the leading institute. The *Journal of Endodontics* was the leading journal, while authors JNR Martins and G Gambarini produced the highest number of papers.

**Conclusions:**

This bibliometric analysis demonstrated that scientific publications have increased tremendously since 2008. Significant contributions have been made by developing and developed nations. The *Journal of Endodontics* and Jazan University have been identified as the leading journal and institute.

## 1 BACKGROUND

The morphology of roots and their canals is quite complex, and sufficient knowledge regarding this morphology is an essential facet required for planning surgical and endodontic interventions (Iqbal et al., 2022). Variations in roots and the canals' curvatures (angulation, length, diameter, radius, level, and height) can make it difficult to access, clean, and treat the root canals, which can lead to complications such as infection and pain encountered in dentistry on a daily basis, which can become challenging for the dentist (Ozcan et al., 2016). Thorough debridement and obturation of the root canal system are therefore pivotal for successful endodontic treatment (Karobari, Noorani, et al., 2021). The presence of microorganisms and pulp tissue (necrotic as well as healthy) in the root canals can be adequately accessed and treated during endodontic management: if sound awareness regarding the morphology and anatomy of the root canal and its associated complexities is undertaken, proper root‐ends management can be achieved, including the following conditions: adequate access to the root canals, thorough debridement of the root canals, and effective obturation of the root canals (Estrela et al., 2014). Likewise, uncomplicated surgical removal of the tooth is also efficiently dealt with if a clear visual representation of the tooth's root curvature and any periapical pathologies are considered before surgery (Rodrigues et al., 2021).

Root and canal morphologies have been found to vary substantially around the globe between different communities, within different communities, and even in the same individuals (Karobari, Arshad, et al., 2022; Karobari, Assiry, et al., 2023; Karobari, Parveen, et al., 2021). Therefore, the availability of pre‐evaluation records, such as radiographs, cephalograms, and other imaging techniques, before the commencement of treatment involving the tooth's root will enable the dental specialist to plan a suitable management approach for their patients, a consequence of the root canal deviations (AAE and AAOMR Joint Position Statement, 2015). Periapical radiographs, microcomputed tomography (μCT), and dental panoramic tomography had been employed previously to visualize the curvature of root canals (Hartmann et al., 2019). However, the radiological advancements in the field of healthcare and especially dentistry, with the development of cone‐beam CT (CBCT), magnetic resonance imaging, and CT, provide a three‐dimensional (3D) visualization of oral structures and their anatomical variations (Abu‐Melha, 2021).

CBCT is an advanced imaging technique that can produce a more accurate and detailed visualization of the root and canal morphology in three different planes, that is, sagittal, coronal, and axial planes, than traditional two‐dimensional (2D) radiographs, which can help the dentist to plan and execute more successful endodontic treatments (Demirbuga et al., 2013). The CBCT imaging has served to subdue the limitations experienced in conventional radiographs, likely the 2D view, distortions and superimpositions of other anatomic structures, and so forth (Karobari, Iqbal, et al., 2023; Ozcan et al., 2016). CBCT has also gained recognition as a cost‐effective and dose‐effective imaging modality for diagnostic evaluations (Larheim et al., 2015). Research by Alamri et al. (2012) summarized the application of CBCT in dental fields such as restorative and endodontics, oral and maxillofacial surgery, periodontics, orthodontics, prosthodontics, and even forensic dentistry.

Scientiometry employs a quantitative means to study the scientific influence of a publication based on the number of citations (Blakeman, 2018; Moed, 2009). Bibliometric analysis is a comprehensive method of attaining evidence that combines science with statistical methods and enables quantitatively analyzing information (Moodley et al., 2015). In addition, the impact and growth of scientific research, progress within specific fields, and ongoing trends can be identified (Moodley et al., 2015). It further allows scholars to identify the main study domains and identification of prominent authors, research trends, leading journals, institutes, and authors in a particular domain (Wu et al., 2017). Bibliometric analysis has been extensively conducted in various domains like minimally invasive dentistry, periodontal regeneration, salivary biomarkers, dental polymers, and endodontic microbiology (Alam et al., 2021, 2022; Ali et al., 2023; Karobari, Maqbool, et al., 2021; Shaikh et al., 2019).

Hence, this study aimed to identify the publication trends, leading countries, authors, institutes, most highly cited scientific publications, and commonly used keywords concerning root and root canal morphology using CBCT.

## 2 METHODOLOGY

### 2.1 Search strategy

The search for relevant papers was performed on September 25, 2023 using the Scopus database by Elsevier. All the relevant papers were searched from 2008 to March 2023 to execute the process of bibliometric analysis. This electronic search included the research domain; beside this, it also comprised the titles, abstracts, and associated keywords. To identify appropriate results from the database, the keywords titled “root and root canal morphology using CBCT” were used to search.

### 2.2 Inclusion and exclusion criteria

The inclusion criteria comprised:

a) Papers written in English language only.
b) “Original Article” and “Reviews” as the type of publication.
c) Dentistry as the domain.

**Figure 1:**
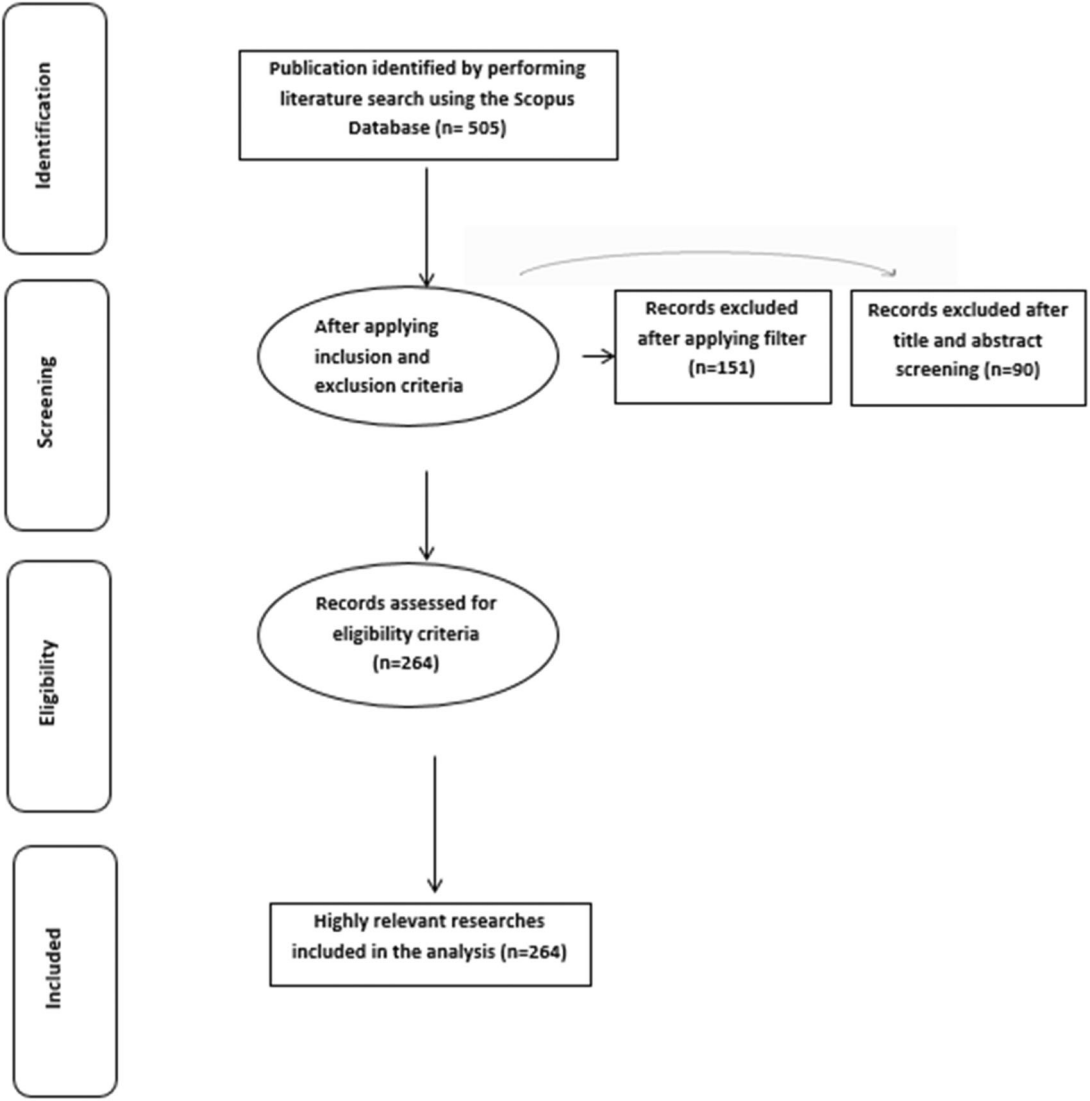
Four‐phase flow diagram of data extraction and filtration process of publications related to root and root canal morphology using the CBCT. CBCT, cone‐beam computed tomography.

The exclusion criteria included:

a) Publications not aiming at the topic.
b) Other types of publications, which included case studies, chapters from books, and thesis.
c) Publications from other domains (Figure 1).

### 2.3 Data analysis

After performing the search and scrutinizing the relevant papers, all the selected articles were downloaded from the Scopus database using the comma‐separated values format. This file was saved and analyzed using VOSviewer (v1.6.17; Center for Science and Technology Studies, Leiden University), a bibliometric software program (van Eck & Waltman, 2010; Najmi et al., 2023; Shamszadeh et al., 2019). Results were downloaded from Vosviewer and were further evaluated on Microsoft Excel as tab‐delimited files.

VOSviewer software can create collaborative linkages among several variables and keywords. The sizes of the bubbles within the created maps designate the number of scientific publications, whereas the distance in the middle of the two bubbles specified the connection and relationship between the two items. The color of each bubble had different significance within individual visualization. Keywords with the highest number of occurrences were carefully chosen and resulted in generating visualization maps.

## 3 RESULTS

**Figure 2:**
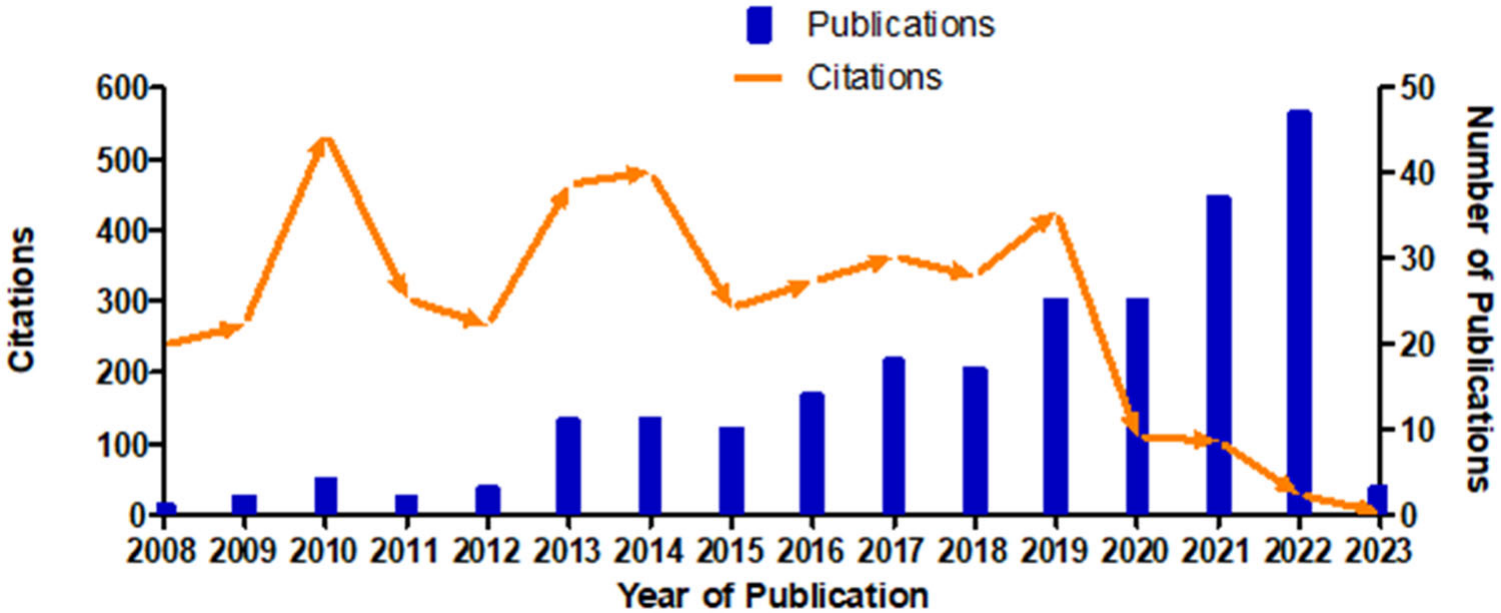
Scientific publications and citations trend from 2008 to 2023.

Figure 2 illustrates the total number of scientific publications and the citations attained. It can be visualized that the first paper was published in 2008 and attained 238 citations. Over the period, the number of publications increased gradually, while at the same time, a significant increase in citations was identified. After 2017, the number of research articles (*n* = 18) increased; in 2020, *n* = 25 papers were published. This trend continued increasing with *n* = 37 and *n* = 47 publications in 2021 and 2022, respectively. Similarly, the trend of publications continued during the year 2023, with a total of 34 publications, and this number is expected to increase by the end of the year.

Regarding the number of citations, the highest number of citations was seen during the year 2010. During this year, *n* = 4 papers were published, which received 536 citations. Following this, a reduction in the number of citations could be analyzed during 2012; however, the number of publications increased again in 2014, with 11 publications, with 482 citations. Similarly, the trend continued until 2019, when 25 papers attained 426 citations. The publication trend has continuously increased over the years, but the number of citations has decreased since 2020.

Table 1 depicts the leading countries that have produced the most papers in this field. Saudi Arabia, India, and China published the most papers, *n* = 40, *n* = 39, and *n* = 24, respectively. Brazil, Iran, and the United States have also made significant contributions in this field.

**Table 1:** Leading countries.

Country	TP	TC	TLS
United States	20	1058	220
China	24	823	206
Brazil	23	670	139
India	39	607	143
South Korea	9	356	82
Turkey	13	333	99
Iran	23	292	127
Italy	9	244	88
Saudi Arabia	40	240	156
Portugal	6	211	74

Abbreviations: TC, total citations; TLS, total link strength; TP, total publications.

Concerning citations, the United States attained the highest number of citations (*n* = 1058), followed by China (*n* = 823) and Brazil (*n* = 670). Despite publishing the highest number of papers in this domain, Saudi Arabia and India received lesser citations (Table 1).

**Figure 3:**
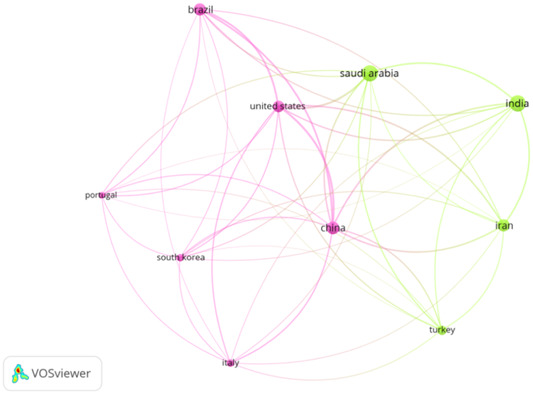
Leading countries.

**Figure 4:**
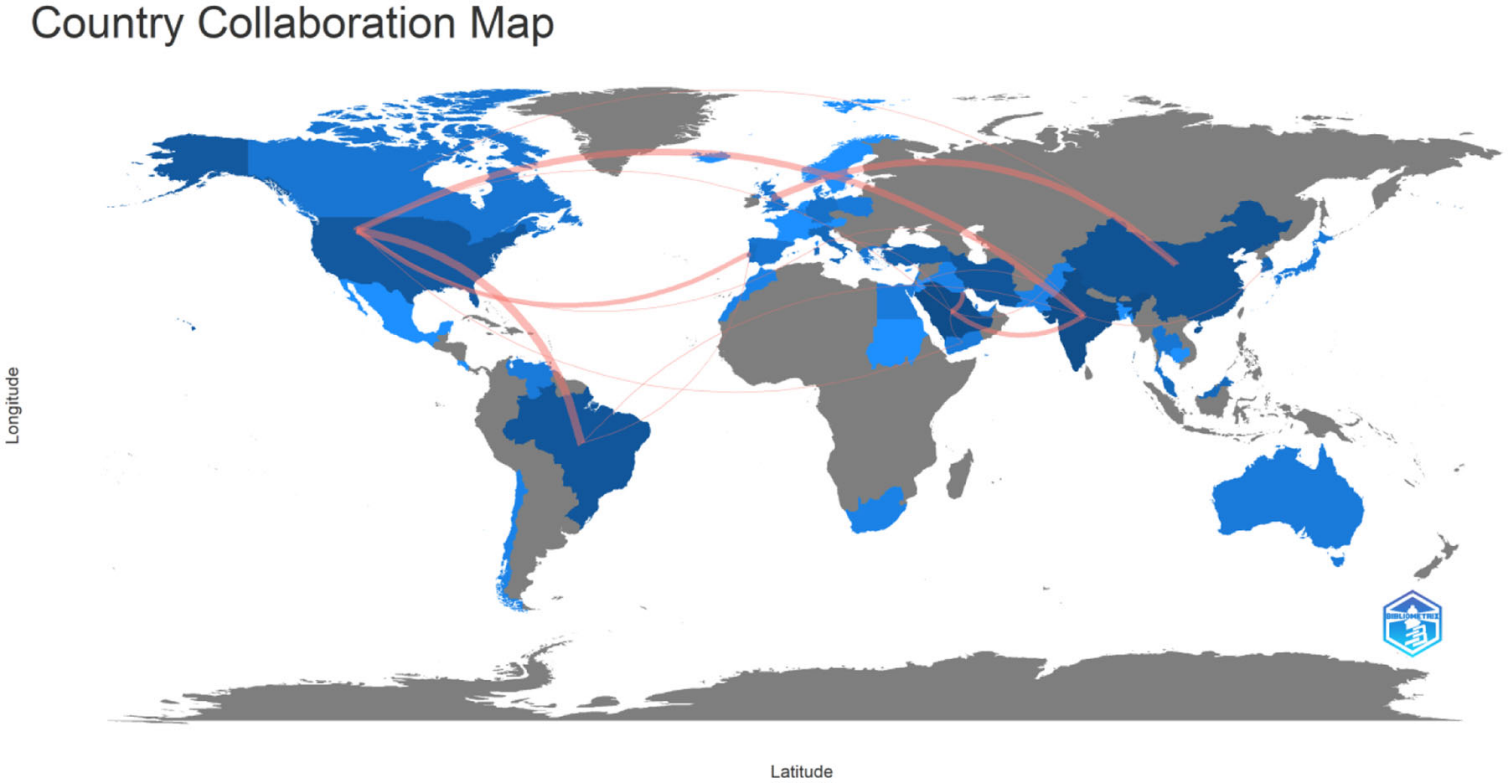
Countries making significant contribution.

Figures 3 and 4 describe the collaborative network of countries that have published the most papers in this domain.

Table 2 demonstrates the leading organization which made significant contributions in this domain. Jazan University, from Saudi Arabia, published the most papers (*n* = 6); however, these papers only received *n* = 76 citations. Interestingly, all the other universities highlighted in the table published *n* = 2 papers each. Amongst them, Cardiff University, Sichuan University and the University of Washington received the highest number of citations (Table 2).

**Table 2:** Leading organizations.

Organization	Country	TP	TC	TLS
Cardiff University	United Kingdom	2	229	10
Sichuan University	China	2	224	7
University of Washington	United States	2	183	7
Sifa University	Turkey	2	87	4
Jazan University	Saudi Arabia	6	76	4
Universidade De Lisboa	Portugal	2	60	7
University of Malaya	Malaysia	2	52	1
Chulalongkorn University	Thailand	2	50	2
Shahid Beheshti University of Medical Sciences	Iran	2	46	5
Sapienza University of Rome	Italy	2	39	1

Abbreviations: TC, total citations; TLS, total link strength; TP, total publications.

**Figure 5:**
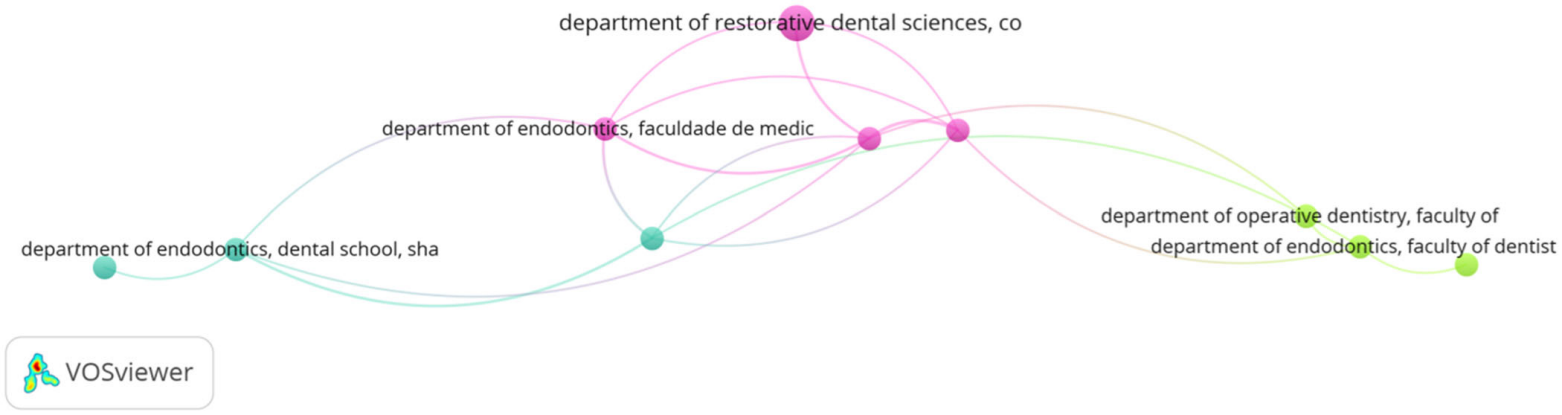
Collaborative network of author's publications from different organizations.

Total link strength (TLS) depicts the level of association and cooperation amongst various countries. In this aspect, universities such as Cardiff, Sichuan, University of Washington, and Universidade De Lisboa displayed the highest research association in this field. Figure 5 identifies the collaborative nature of research publications by the authors in those organizations.

**Figure 6:**
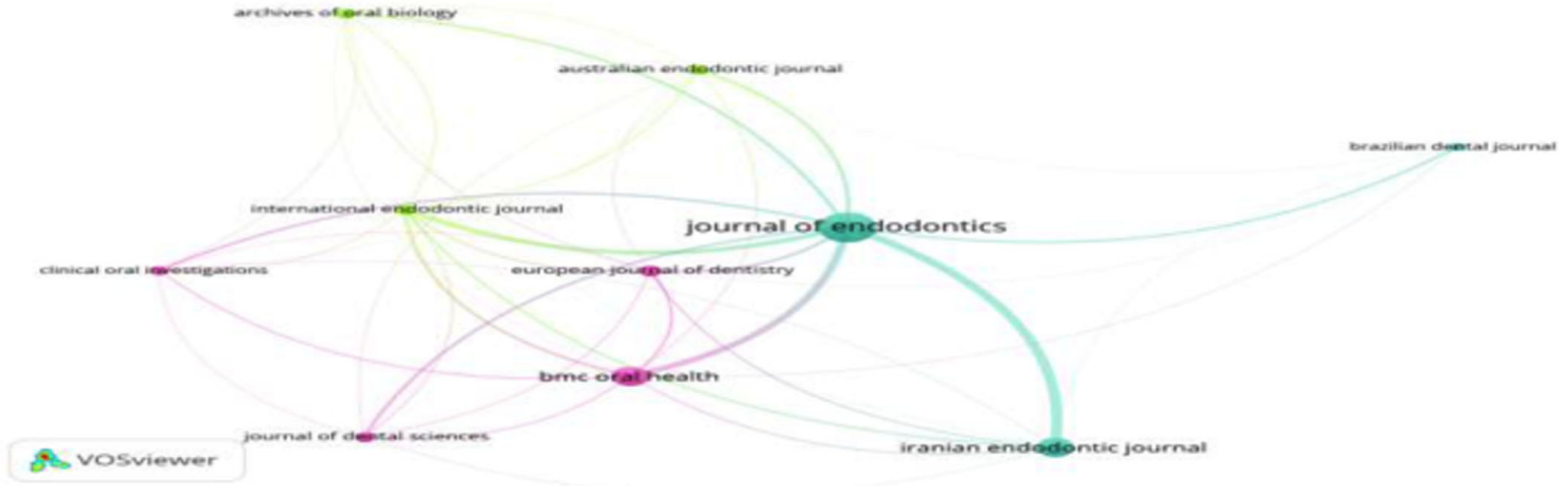
Leading journals.

Table 3 identifies the top journals that published the maximum number of papers in this field. *Journal of Endodontic* printed the highest number of papers in this domain. *Iranian Endodontic Journal* and *BMC Oral Health* ranked second by publishing *n* = 17 papers each, followed by the *European Journal of Dentistry*, which printed *n* = 8 papers (Figure 6).

**Table 3:** Leading journals.

Journal	TP	TC	Country	Publisher	IF	Q	TLS
*Journal of Endodontics*	32	2097	USA	Elsevier	4.422	1	207
*International Endodontic Journal*	7	508	UK	Wiley‐Blackwell Publishing Ltd	5.165	1	79
*European Journal of Dentistry*	8	269	Turkey	Georg ThiemeVerlag	0	1	51
*Iranian Endodontic Journal*	17	244	Iran	Iranian Center for Endodontic Research	0	3	77
*Clinical Oral Investigations*	6	169	Germany	Springer Verlag	3.734	1	34
*BMC Oral Health*	17	135	UK	Biomed Central Ltd	3.73	1	86
*Journal of Dental Sciences*	7	80	China	Association for Dental Sciences of The Republic of China	3.719	2	35
*Brazilian Dental Journal*	5	77	Brazil	Associacao Brasileira De Divulgacao Cientifica	0	2	17
*Australian Endodontic Journal*	7	63	USA	Wiley‐Blackwell	2.259	2	30
*Archives of Oral Biology*	7	58	UK	Elsevier	2.64	2	28

Abbreviations: TC, total citations; TLS, total link strength; TP, total publications.

Regarding the number of citations, the *Journal of Endodontic* attained the highest (*n* = 2097), followed by the *International Endodontic Journal* and *European Journal of Dentistry*, which attained *n* = 508 and *n* = 269 citations, respectively. Furthermore, most of the journals in the list were Q1, while the *Journal of Dental Sciences*, *Brazilian Dental Journal*, *Australian Endodontic Journal*, and *Archives of Oral Biology* belonged to Q2; only the *Iranian Endodontic Journal* was categorized as Q3. Concerning impact factor, *International Endodontic Journal* had the highest impact factor (5.165), followed by the *Journal of Endodontics* (4.422). At the same time, the *Journal Clinical Oral Investigations* and *BMC Oral Health* had an impact factor (3.73), respectively.

**Figure 7:**
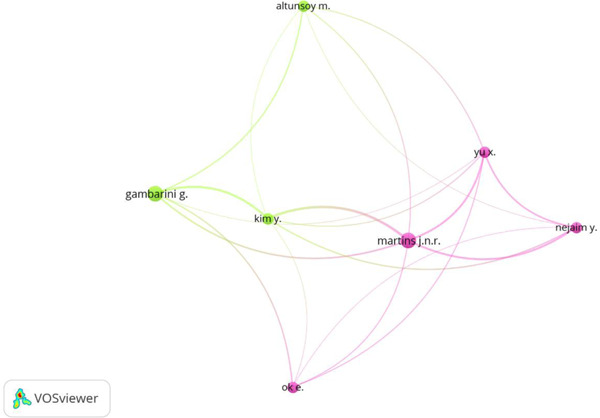
Leading authors.

Table 4 depicts the leading authors who have published the highest number of papers concerning root and root canal morphology using CBCT. Authors JNR Martins and G Gambarini have published *n* = 6 papers each, while all the other authors in the list published *n* = 4. Author Yu X had the highest number of citations, followed by Y Kim and Yuri Najaim, who attained *n* = 294 and *n* = 236 (Table 4). Figure 7 identifies authors which made contributions significantly in this field.

**Table 4:** Highly prolific authors.

Author	TP	TC	TLS
Yu X	4	380	16
Y Kim	4	294	20
Yuri Nejaim	4	236	14
JNR Martins	6	211	25
G Gambarini	6	189	17
Mustafa Altunsoy	4	173	9
Evren Ok	4	173	9

Abbreviations: TC, total citations; TLS, total link strength; TP, total publications.

Table 5 describes the top highly cited publications on root and root canal morphology using CBCT. Papers by Matherne RP published in 2008, had the highest citations (*n* = 238), (Matherne et al., 2008) followed by two papers by Neelakantan in 2010 (Neelakantan, Subbarao, Ahuja, et al., 2010; Neelakantan, Subbarao, & Subbarao, 2010). Interestingly, most of these highly cited scientific articles were printed by the *Journal of Endodontics* and the *International Endodontic Journal*.

**Table 5:** Highly cited publications.

Paper	Author	Citations	Journal	Year	Links
Use of cone‐beam computed tomography to identify root canal systems in vitro.	Matherne RP	238	*Journal of endodontics*	2008	6
Comparative evaluation of modified canal staining and clearing technique, cone‐beam computed tomography, peripheral quantitative computed tomography, spiral computed tomography, and plain and contrast medium–enhanced digital radiography in studying root canal morphology.	Neelakantan P	206	*Journal of endodontics*	2010	2
Cone‐beam computed tomography study of root and canal morphology of maxillary first and second molars in an Indian population.	Neelakantan P	189	*Journal of endodontics*	2010	3
Morphology of maxillary first and second molars analyzed by cone‐beam computed tomography in a Korean population: variations in the number of roots and canals and the incidence of fusion.	Kim Y	170	*Journal of endodontics*	2012	1
Analysis of the internal anatomy of maxillary first molars by using different methods.	Baratto Filho F	162	*Journal of endodontics*	2009	1
Use of CBCT to identify the morphology of maxillary permanent molar teeth in a Chinese subpopulation.	Zhang R	154	*International endodontic journal*	2011	2
Use of cone‐beam computed tomography to evaluate root and canal morphology of mandibular molars in Chinese individuals.	Zhang R	149	*International endodontic journal*	2011	3
Detection of permanent three‐rooted mandibular first molars by cone‐beam computed tomography imaging in Taiwanese individuals.	Ming‐Gene Tu	105	*Journal of endodontics*	2009	2

### 3.1 Keywords

**Figure 8:**
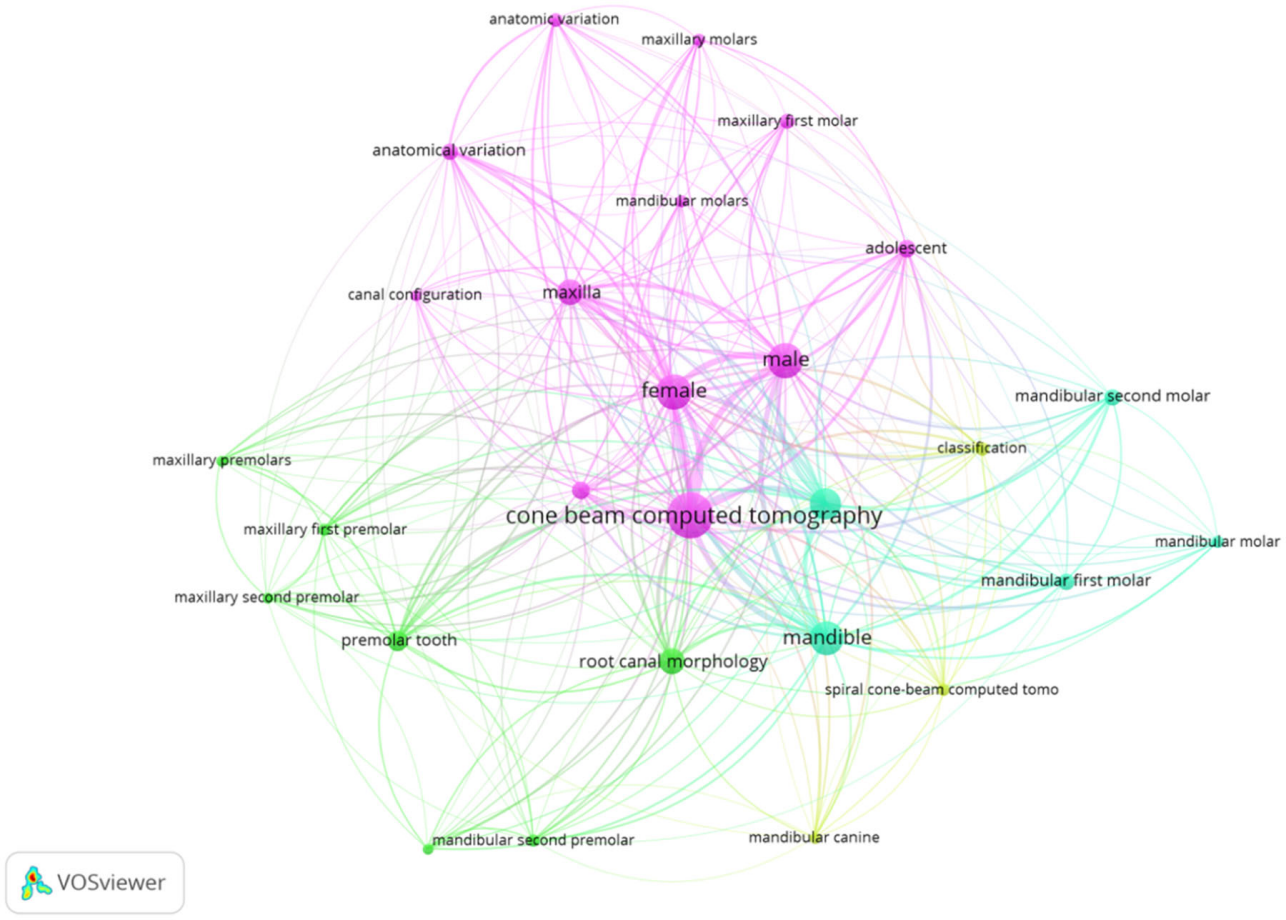
Commonly used keywords.

Figure 8 identifies researchers' keywords with the highest number of cooccurrences. Keywords which were pertinent to the topic and repeated 10 times were encompassed. Among these 665 keywords, 70 were included (were used in at least 10 publications). The most frequently occurring keywords included “cone‐beam computed tomography” (TLS = 2430; occurrence rate = 160 papers), “root canal morphology” (TLS = 510; occurrence rate = 53 articles), “diagnostic imaging” (TLS = 1320; occurrence rate = 72 papers), “premolar tooth” (TLS = 505; occurrence rate = 33 papers), and “mandibular first molar” (TLS) = 334; occurrence rate = 20 papers) (see Figure 8).

**Figure 9:**
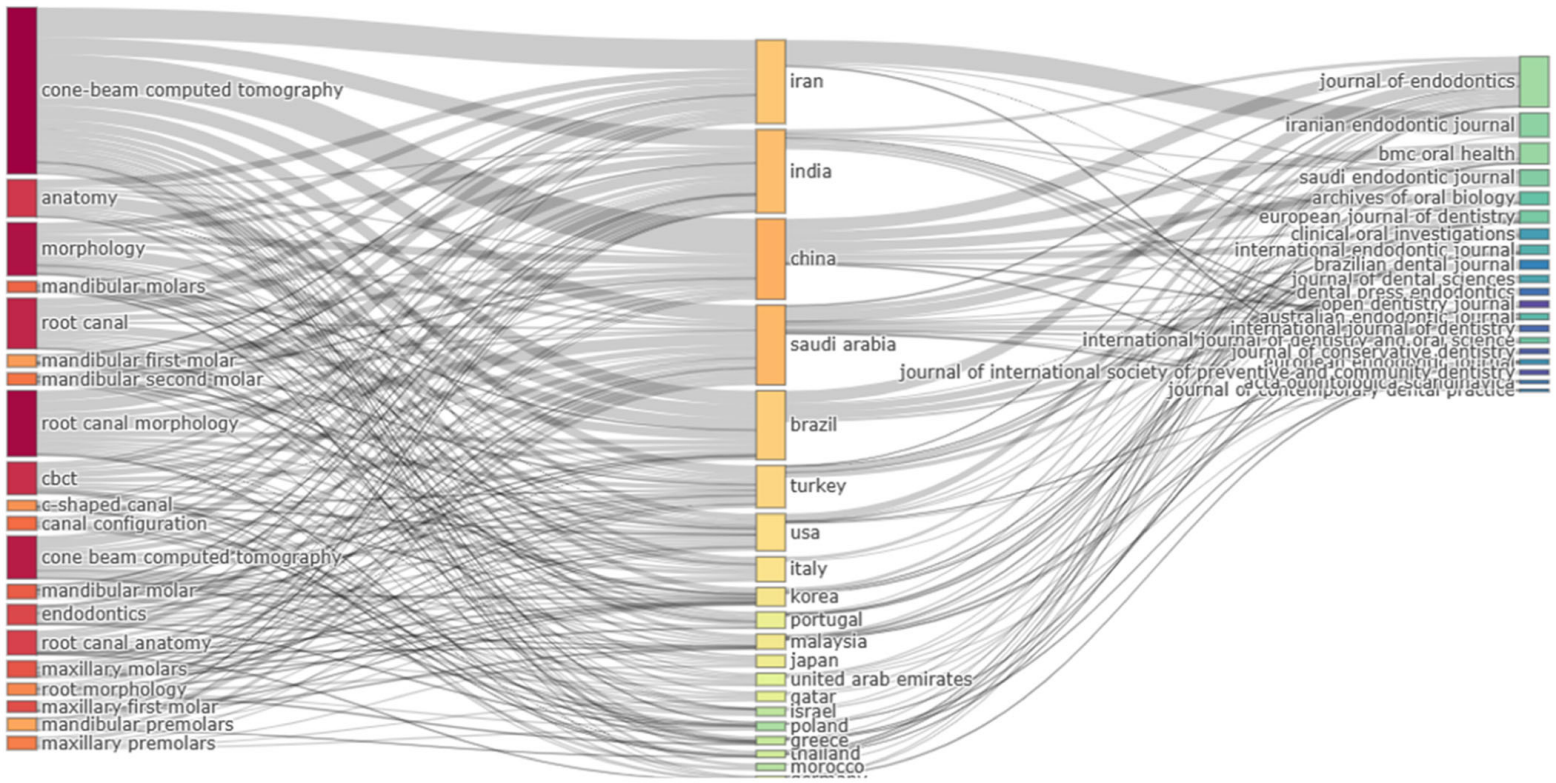
Three‐factor plot displaying the relationship among keywords (right), countries (middle), and journal (left) focusing on root canal morphology.

Figure 9 show that researchers from different countries published their publications in certain journals using specific keywords. It can be seen that scholars from the leading countries (Iran, India, China, Saudi Arabia, Brazil, and the United States) preferred to publish their work in journals which included “*Journal of Endodontics, Iranian Endodontic Journal, BMC Oral Health, Saudi Endodontic Journal, International Endodontic Journal*” and most repeatedly keywords included “cone‐beam computed tomography,” “root canal morphology,” and “morphology.”

**Figure 10:**
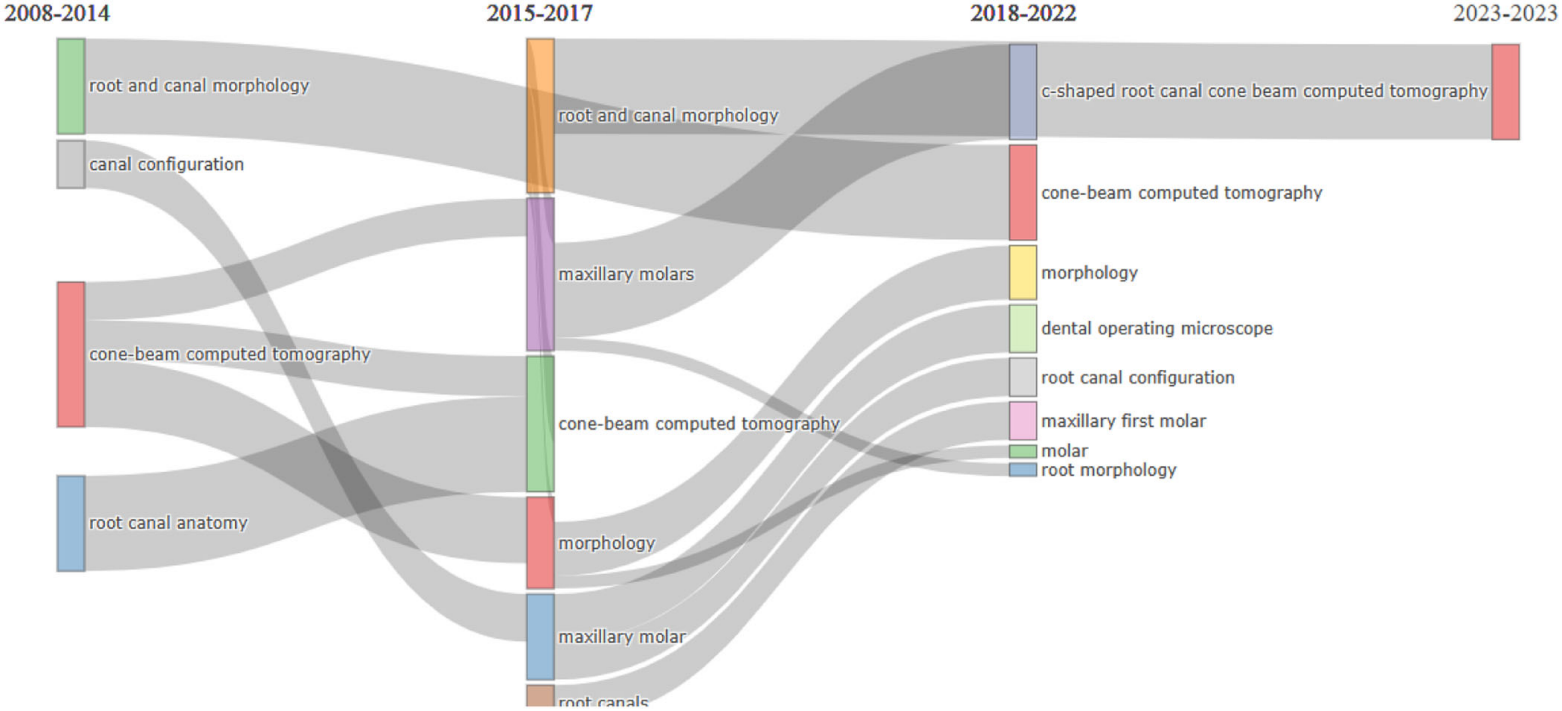
Thematic evolution of keywords.

Figure 10 illustrates the evolution of keywords over time from 2008 to 2023. The keywords that were used in this research has been highlighted (generated using Biblioshiny).

## 4 DISCUSSION

Bibliometrics is a tool that identifies the trends in scientific research; it allows for the analysis of data using mathematical and statistical methods to generate voluminous information. This tool can quantify the impact of scientific publications and the contributions made by authors, journals, countries, and organizations and identify the keywords that are used most often (Jiang et al., 2021; Karobari, Maqbool, et al., 2021; Yu & Chang, 2022). Current bibliometric analysis aimed to detect the scientific publications, productive authors, leading nations, journals, and organizations which produced publications focusing on root and root canal morphology evaluated with CBCT.

### 4.1 The trend in scientific publications

The trend of yearly research publication and citations attained shows that the first paper was published in 2008 by Matherne RP in the *Journal of Endodontics*. In this paper, extracted teeth were subjected and exposed to CBCT, charged coupled device (CCD), and digital radiography. The study found the beneficial role of CBCT in identifying root canal systems. This paper has received 238 citations (Matherne et al., 2008). Another publication which had received the highest citations was published by Neelakantan P et al. in 2010. This paper attained 206 citations and was published in the *Journal of Endodontics* (Neelakantan, Subbarao, & Subbarao, 2010). Furthermore, it was identified that, with time interest of researchers in this domain increased significantly with time, this could be identified by increase in number of publications in the year 2023, with the number expected to increase in future.

### 4.2 Leading countries

Findings from the current analysis depict that Saudi Arabia made a substantial contribution by producing the highest number of publications in this area. These rich contributions made by Saudi Arabia could be attributed to the 2030 Vision plan executed, in which the country aimed to be identified as one of the top 10 countries concerning the Global Competitiveness Indicator. One of the factors was to gain a high ranking by increasing the number of scientific publications; hence, many research grants and funding have been allocated for research (Yu & Chang, 2022). In addition, researchers from India and China also made significant contributions.

Interestingly, regarding the citations attained, the scientific publications from the United States attained the highest number of citations, despite publishing fewer papers comparatively. These outcomes are consistent with the earlier bibliometric analysis, which identified the significant role of the National Institute of Dental and Craniofacial Research in the United States, which provides finance for research (Alam et al., 2021; Qasim et al., 2021).

### 4.3 Leading journals

Findings from the current study reveal that the highest number of papers were published by leading journals, having a high impact factor. The impact factor showed the greater influence of these research journals and the significant impact scientific papers would have after publication (Sharma et al., 2014). However, the journal's impact factor, recognized as an indicator for the journal performance indicator, has been ascertained as ineffective means for evaluating the quality of scientific publications (Casadevall & Fang, 2015; Garfield, 2006). Nevertheless, in the current analysis *Journal of Endodontics*, the *International Journal of Endodontics* has been identified as the leading Journal. In addition, seven journals in the list had a high impact factor, while five had a Q1 ranking. Most journals were published in the United Kingdom, the United States, and Germany. However, it was also identified that journals from Brazil, Turkey, and Iran also made significant contributions. This indicates that aside from contributions from developed countries, significant contributions are being made from developing nations.

Citation analysis is another practical approach used in bibliometric analysis to identify the research productivity and the impression of researchers, institutions, articles, and journals. Despite its comprehensive use, the outcomes achieved by the citation analysis have been a source of frequent discussion, signifying that the number of citations does not automatically reveal the scientific impact of a scientific publication (Seglen, 1998; Whitehouse, 2001).

### 4.4 Leading authors

Significant contributions made by authors have been highlighted in the current analysis. Author JNR Martin and Gambarini G, associated with Universidade de Lisboa and the University of Rome, published the highest number of papers in this domain. Regarding the number of citations attained, author Yu X associated with Jinan Key Laboratory of Oral Tissue Regeneration, received the highest number of citations in China.

### 4.5 Highly cited publications

The first paper which received the highest number of citations was published by Matherne RP in the *Journal of Endodontics* in 2008. In this study, extracted teeth were exposed to CBCT, a photostimulable phosphor plate, and CCD to analyze the number of root canal systems. CBCT proved successful in identifying the root canals as compared to other devices (Matherne et al., 2008).

In the Journal of Endodontics, the second paper which attained the highest number of citations was published by Neelakantan P in 2010. This publication evaluated the accuracy of CBCT, quantitative CT, spiral CT, and plain and contrast digi to evaluate the morphology of the root canal. The canals of the extracted teeth were stained, and clearing techniques were performed. This study concluded CBCT and Pqct as accurate tools for identifying the root canal system (Neelakantan, Subbarao, & Subbarao, 2010).

The third significantly cited paper was also authored by Neelakantan P in 2010 and published by the *Journal of Endodontics*. This paper evaluated the number and morphology of the root canals within the Indian participants with the help of CBCT. The paper concluded the valuable role of CBCT in detecting morphology (Neelakantan, Subbarao, Ahuja, et al., 2010).

Kim et al. (2012) authored the fourth paper in 2012 and published it in the *Journal of Endodontics*. This research aimed to determine the alignment and number of roots and canals aligned with Vertucci's classification in the Korean participants. This research identified various rare features unidentified as yet in other populations.

Baratto Filho et al. (2009) published the fifth paper in the *Journal of Endodontics* in 2009. This paper explored the anatomy and morphology of the maxillary first molar with the help of ex vivo assessment carried out by operating microscope, clinical analysis, and CBCT. These methods helped identify the number of extra root canals, positions, foramina number, and frequency of canals that remained undetected were evaluated. The study confirmed the usefulness of CBCT and operating microscope in detecting the morphology of the first molar.

### 4.6 Keywords

The findings from the current analysis presented that keywords helped to determine the trend of scientific publications in this domain. Keywords help identify the exact scientific literature required, and at the same time, they work as “coded message,” which generates the preferred research literature (Najmi et al., 2023; Natarajan et al., 2010). Therefore, it is important to choose keywords that can easily help identify the relevant literature (Arshad et al., 2020). keywords with the highest number of cooccurrences from the current analysis would support researchers and investigators in identifying articles pertinent to root canal morphology.

In addition, the three‐factor relationship showed that the keyword “cone‐beam computed tomography” was generally used by authors from Iran, India, and China. This keyword seemed quite generic, yet, it was used recurrently. However, less attention was given to the name of teeth when used as keywords. The keywords' thematic evolution occurred during four different phases; 2008−2014, 2015−2017, 2018−2022, and 2023−2023. It was seen that keywords like “cone‐beam computed tomography,” and “root canal morphology” had been constantly used.

### 4.7 Limitations

The main limitation associated with bibliometric analysis is that only the Scopus database was used for analysis. Publications available on other databases, such as Google Scholar and Web of Science, may have been missed. The number of citations also tends to vary amongst different databases. Older publications, due to more time, receive higher citations. Second, self‐citations by authors were not evaluated as there were no available means for identifying them.

## 5 CONCLUSION

This bibliometric analysis demonstrated that scientific publications have increased tremendously since 2008. Significant contributions have been made by developing as well as developed nations. *Journal of Endodontic* and Jazan University was the leading Journal and institute. Authors JNR Martins and G Gambarini made significant contributions to this field.

## AUTHOR CONTRIBUTIONS

Conceptualization: Mohmed Isaqali Karobari and Beenish Fatima Alam. *Methodology*: Mohmed Isaqali Karobari, Beenish Fatima Alam, and Tahir Yusuf Noorani. *Formal analysis and investigation*: Beenish Fatima Alam, Raima Bashir, Muhammed Faisal Fahim, and Mubashir Baig Mirza. *Writing—original draft preparation*: Mohmed Isaqali Karobari, Beenish Fatima Alam, Mubashir Baig Mirza, and Tahir Yusuf Noorani. *Writing—review and editing*: Mohmed Isaqali Karobari and Tahir Yusuf Noorani. All authors read and approved the final manuscript.

## CONFLICT OF INTEREST STATEMENT

The authors declare no conflict of interest.

## Data Availability

The data sets used and/or analyzed during the current study are available from the corresponding author upon reasonable request.
